# Multicentre, 2×2 factorial, randomised, open-label trial of neuromodulators and cough control therapy for refractory or unexplained chronic cough: the FORTITUDE trial protocol

**DOI:** 10.1136/bmjopen-2026-126545

**Published:** 2026-09-18

**Authors:** Elena Kum, Wafa Hassan, Sue Beaudin, Geoff Strong, Kim Smith, Ana Oliveira, Mustafa Al-Saiedy, Renata Mancopes, Ana Sofia Grave, Melanie Bernier, Anurag Bhalla, Danica L Brister, Andréanne Côté, Simon Couillard, Stephen K Field, Faye Johnston, Robert Newman, Nada Popovic, Alan G Kaplan, Dina Brooks, Gordon Guyatt, Imran Satia

**Affiliations:** 1 Department of Medicine, McMaster University, Hamilton, Ontario, Canada; 2 Department of Health Research Methods, Evidence, and Impact, McMaster University, Hamilton, Ontario, Canada; 3 Respiratory Research and Rehabilitation Laboratory (Lab3R), School of Health Sciences (ESSUA), University of Aveiro, Aveiro, Portugal; 4 Department of Medicine, University of Calgary, Calgary, Alberta, Canada; 5 Swallowing Rehabilitation Research Laboratory, KITE Research Institute, Toronto, Ontario, Canada; 6 Rehabilitation Institute, University Health Network, Toronto, Ontario, Canada; 7 Department of Medicine, Laval University, Québec City, Quebec, Canada; 8 Department of Medicine, Western University, London, UK, Canada; 9 Faculté de médecine et des sciences de la santé, Universite de Sherbrooke, Sherbrooke, Quebec, Canada; 10 Chronic Cough Canada, Hamilton, Ontario, Canada; 11 Family Physicians Airway Group of Canada, Markham, Ontario, Canada; 12 Respiratory Medicine Special Interest Focus Group, College of Family Physicians of Canada, Mississauga, Ontario, Canada; 13 School of Rehabilitation Science, McMaster University, Hamilton, Ontario, Canada; 14 Firestone Institute for Respiratory Health, St Joseph’s Healthcare Hamilton, Hamilton, Ontario, Canada

**Keywords:** Randomized Controlled Trial, Pulmonary Disease, Clinical Protocols

## Abstract

**Introduction:**

While clinicians commonly prescribe neuromodulators for the treatment of refractory or unexplained chronic cough, no trials have directly compared these treatments. Furthermore, although guidelines recommend non-pharmacological management involving physiotherapy and speech-language therapy, few trials have evaluated its effectiveness.

**Methods and analysis:**

FORTITUDE (*F*act*O*rial *R*andomized *T*r*I*al *T*o eval*U*ate neuromo*D*ulators and cough control th*E*rapy) is a 6-week, 2×2 factorial, randomised, open-label trial with an observational extension up to 1 year. The trial will include Canadian hospital sites enrolling 124 patients. Patients ≥18 years old with either refractory or unexplained chronic cough will undergo independent 1:1 randomisation to pharmacotherapy—either low-dose morphine (up to 5 mg two times per day) or pregabalin (up to 150 mg two times per day)—and concurrent cough control therapy (CCT) or no CCT. The CCT will consist of five virtual sessions delivered over 6 weeks by a trained physiotherapist or speech-language pathologist. Change from baseline in 24-hour cough frequency at 6 weeks represents the primary outcome. Secondary outcomes include change from baseline in awake, sleep and cough bout frequencies; cough severity on the McMaster Cough Severity Questionnaire and the 100-mm Visual Analogue Scale; and cough quality of life on the Leicester Cough Questionnaire at 6 weeks. We will analyse safety and tolerability at 6 weeks and explore potential interactions between neuromodulators and CCT. Follow-up in an observational extension of up to 12 months post-randomisation will assess long-term treatment patterns, adherence and safety in a real-world setting.

**Ethics and dissemination:**

Health Canada’s Pharmaceutical Drugs Directorate has provided approval to conduct this study. We have approval from the Hamilton Integrated Research Ethics Board of the Main Coordinating Site, with ethics pending at other participating sites. Results of the main trial and the observational extension will be published as manuscripts in peer-reviewed journals.

**Trial registration number:**

NCT07288528.

**STRENGTHS AND LIMITATIONS OF THIS STUDY**

- The 2×2 factorial randomised design enables simultaneous evaluation of low-dose morphine versus pregabalin and cough control therapy (CCT) versus no CCT.
- The multicentre design across Canadian tertiary cough clinics enhances the generalisability of study findings to specialist clinical settings.
- The 6-week randomised treatment period is followed by an observational extension up to 1 year to assess long-term outcomes.
- The observational extension phase is not randomised, limiting the ability to make causal inferences about treatment groups beyond the 6-week randomised treatment period.
- The study is powered to detect the main effects of morphine versus pregabalin and CCT versus no CCT but is not powered to detect interactions between the two interventions.

## Introduction

### What is the problem to be addressed?

 Chronic cough lasting >8 weeks is a common health problem. In Canada, it affects 16% of adults aged >45 years, representing one of the highest prevalence rates globally.^1^ Although some patients respond to treatments addressing known causes of cough, approximately 26% have, despite optimal therapy, refractory or unexplained chronic cough (RCC/UCC).^2^

RCC/UCC can lead to substantial physical and psychosocial burden.^3^ Patients frequently experience chest pain, urinary incontinence, syncope, headaches, depression and sleep disturbance.^4 5^ Improving quality of life in these patients requires administration of effective therapies.

### Current management of RCC/UCC

In Canada and many countries globally, there remains no licensed treatments for RCC/UCC. Clinical practice guidelines^6 7^ therefore recommend behavioural cough suppression^8–10^ or off-label use of neuromodulators, including low-dose morphine,^11^ pregabalin^8^ or gabapentin.^12^

Two randomised trials have evaluated low-dose morphine given at 5–10 mg two times per day for 2–4 weeks in adults with RCC/UCC.^11 13^ The first trial in 27 patients found that, compared with placebo, morphine reduced cough severity by 2 points (95% CI 1.1 to 2.1) on a 10-point scale.^11^ The second trial in 22 known responders found that it improved 24-hour cough frequency by 71.8% and Cough Severity on the 100-mm Visual Analogue Scale (CS-VAS) by 27.2 mm compared with placebo.^13^ In both trials, patients generally tolerated morphine, and the most common side effects included constipation and drowsiness.^11 13^

Another neuromodulator, pregabalin, has shown benefit when combined with speech therapy. In a randomised trial of pregabalin (150 mg two times per day) plus speech therapy, the combination improved cough quality of life on the Leicester Cough Questionnaire (LCQ) by 3.5 points (95% CI 1.1 to 5.8) and the CS-VAS by 25.1 mm (95% CI 10.6 to 39.6 mm) compared with speech therapy alone.^8^ Treatment discontinuation occurred in 5% of patients due to drowsiness, nausea or weight gain.

Non-pharmacological interventions that combine education, cough suppression and behavioural modification have also shown benefit.^8–10 14^ In one randomised trial of 87 patients, speech pathology improved cough scores by 2.8 points (95% CI 1.3 to 4.0) on a 5-point scale compared with placebo.^9^ Another randomised trial of 75 patients evaluating physiotherapy with a speech and language therapy intervention (PSALTI) found that, compared with healthy lifestyle advice, PSALTI reduced cough frequency by 41% (95% CI 36% to 95%) and improved LCQ scores by 1.53 points (95% CI 0.21 to 2.85), with effects sustained up to 3 months.^10^ Together, these trials informed a conditional recommendation in favour of non-pharmacological interventions.^6^

### Gaps in the literature

Although current evidence supports the individual efficacy of low-dose morphine^11 13^ and pregabalin,^8^ no head-to-head trials have compared these treatments. The routine use of these agents highlights the need for a large randomised trial directly comparing their benefits and harms.

Furthermore, while guidelines recommend non-pharmacological interventions, randomised trial evidence remains limited to two small studies, with uncertainty regarding durability beyond 3 months,^10^ the specific intervention components that drive benefit, and the factors influencing scalability and broader implementation. This highlights the need for a rigorous evaluation of the effectiveness of a scalable virtual intervention, as well as its fidelity and implementation considerations.

### Why is this trial needed now?

Our trial is the first to evaluate two commonly prescribed neuromodulators—low-dose morphine and pregabalin—and a virtual non-pharmacological intervention called ‘cough control therapy’ (CCT).

We selected low-dose morphine based on evidence from two trials^11 13^ demonstrating cough improvements with doses between 5 and 10 mg two times per day. Although guidelines recommend both pregabalin and gabapentin,^6^ we selected pregabalin due to its more predictable pharmacokinetics and two times per day dosing,^15^ which may improve tolerability and adherence^16^ compared with gabapentin’s three times daily dosing.

Building on work from other groups^8 9 14^ and our own,^17 18^ we developed a structured CCT programme that can be delivered virtually. In a small observational cohort study of patients with RCC/UCC, our group demonstrated the feasibility of virtual CCT and observed a 59.3% reduction in 24-hour cough frequency following the intervention.^18^ We now propose to evaluate virtual CCT in a randomised trial alongside neuromodulators to clarify its independent and additive effects, and assess its acceptability, the components that drive benefit, intervention fidelity and implementation considerations.

## Objectives

The co-primary objectives of FORTITUDE (*F*act*O*rial *R*andomized *T*r*I*al *T*o eval*U*ate neuromo*D*ulators and cough control th*E*rapy) are to compare the effectiveness of (1) low-dose morphine versus pregabalin and (2) virtual CCT versus no CCT in adults with RCC/UCC. Secondary and exploratory objectives include assessment of the safety and tolerability of neuromodulators and CCT, exploration of potential interactions and evaluation of treatment adherence.

## Methods

### Patient and public involvement

Patient partners were involved in the design of the trial and will be involved in the interpretation and reporting of the trial results.

### Trial design

**Figure 1:**
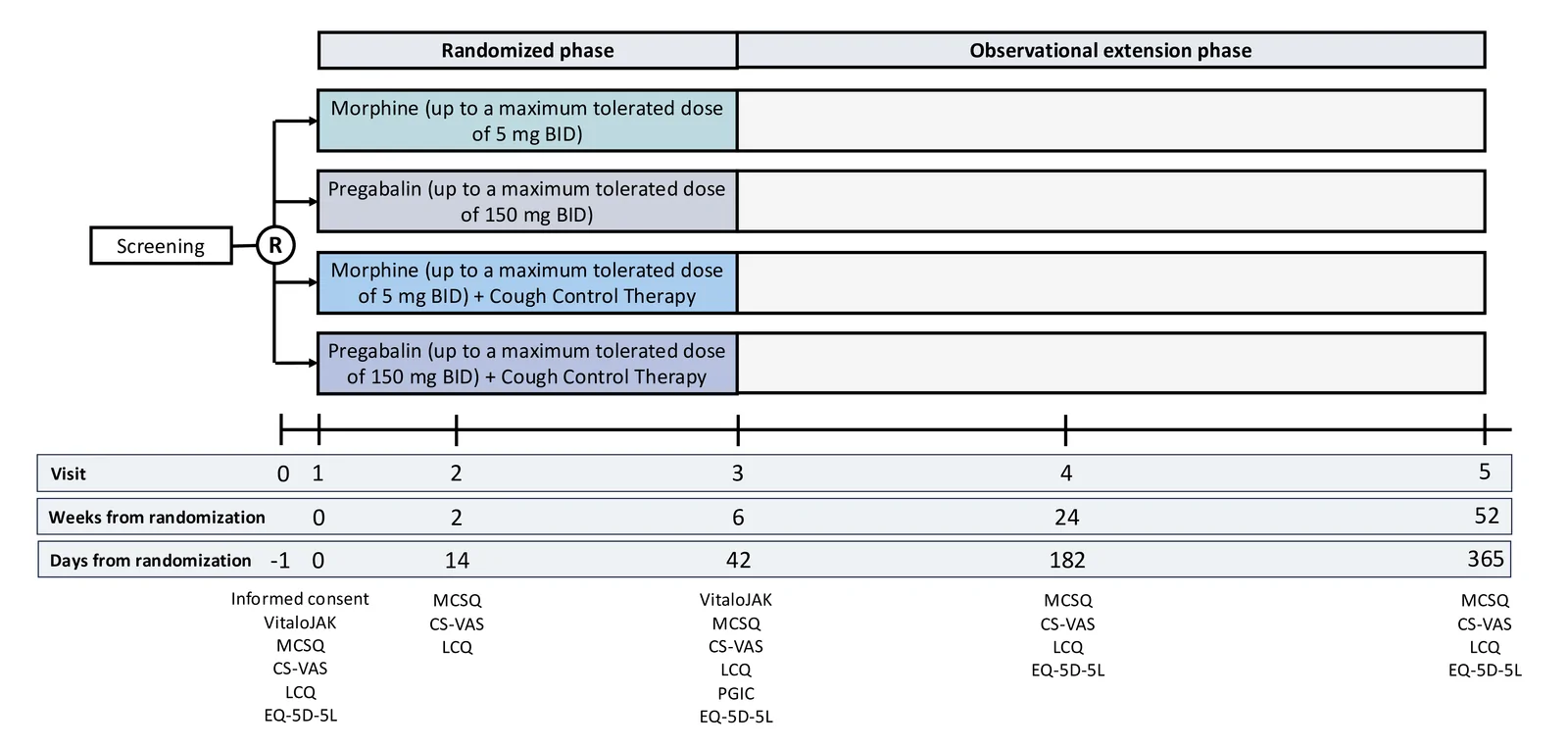
FORTITUDE study design. BID, two times per day; CS-VAS, Cough Severity Visual Analogue Scale; EQ-5D-5L, EuroQol-5 Dimensions-5 Instrument; FORTITUDE, *F*act*O*rial *R*andomized *T*r*I*al *T*o eval*U*ate neuromo*D*ulators and cough control th*E*rapy; LCQ, Leicester Cough Questionnaire; MCSQ, McMaster Cough Severity Questionnaire; PGIC, Patient Global Impression of Change Instrument.

FORTITUDE is a multicentre, superiority, 2×2 factorial, randomised, open-label trial in adults with RCC/UCC (figure 1). Participants will undergo independent 1:1 randomisation to pharmacotherapy (low-dose morphine vs pregabalin) and CCT (CCT vs no CCT), generating four treatment combinations. Primary outcome assessment will occur at 6 weeks.

Following the 6-week randomised period, participants will enter a 1-year observational extension with assessments at 6 and 12 months. Participants will not be required to remain on their randomised treatment, allowing us to examine real-world long-term effectiveness, treatment trajectories, tolerability and CCT uptake.

This trial will adhere to the 2025 Consolidated Standards of Reporting Trials (CONSORT),^19^ the Factorial Randomized Trials Extension^20^ and the 2025 Standard Protocol Items: Recommendations for Interventional Trials (SPIRIT) guidelines.^21^ The trial is registered on ClinicalTrials.gov (NCT07288528). Online supplemental files 1–3 include the Trial Structured Summary, the completed 2025 SPIRIT checklist and the Informed Consent Form. The estimated trial period is September 2026 to September 2029.

### Study setting

FORTITUDE will include five Canadian academic hospital clinics providing secondary or tertiary care to patients with RCC/UCC.

### Screening and informed consent

The study team will approach patients consecutively. Interested patients will receive a study information letter and consent form. Consent discussions may occur in person or by telephone. A research coordinator will obtain written informed consent at an in-person visit.

### Eligibility criteria

Eligible patients will include adults (≥18 years old) with either: (1) RCC, defined as having one or more identifiable causes of cough (asthma, upper airway disease and/or gastro-esophageal reflux disease), but with previous trials of treatment targeting the underlying condition leading to no cough resolution; or (2) UCC, defined as having a cough lasting >8 weeks with (a) a normal chest X-ray/CT in the last 5 years and since the onset of chronic cough; (b) forced expiratory volume in 1 second (FEV_1_) and forced vital capacity (FVC) ≥80% predicted or >lower limit of normal (LLN); (c) FEV_1_/FVC ≥0.7 or >LLN; (d) no evidence of asthma and (e) no regular symptoms of nasal/reflux disease.

Key exclusions include patients who (1) have previously tried low-dose morphine, pregabalin and/or a CCT with full intervention fidelity for the treatment of RCC/UCC; (2) are currently taking morphine or pregabalin for treatment of any indication (ie, RCC/UCC or chronic pain); (3) are currently taking other centrally-acting medication (ie, gabapentin, amitriptyline, dextromethorphan) for the treatment of any indication; (4) have a history of opioid or substance abuse; (5) are a current or ex-smoker with a >20 pack-year history or abstinence <6 months; (6) have cough due to angiotensin-converting enzyme inhibitor use; (7) have symptoms of an upper respiratory infection within the last month that have not resolved; (8) have had an asthma exacerbation within the last month that requires an increase or start of an oral corticosteroid; (9) have other primary pulmonary disorders, including pulmonary embolism, pulmonary hypertension, lung cancer, cystic fibrosis, radiologically proven emphysema, interstitial lung disease or severe bronchiectasis; (10) have creatinine clearance <15 mL/min, including individuals with end-stage renal disease who require haemodialysis or peritoneal dialysis; (11) have participated in another clinical trial of an investigational medicinal product within 30 days or (12) are pregnant or breastfeeding.

### Randomisation, allocation concealment and blinding

Authorised research staff will use an automated centralised randomisation system on Research Electronic Data Capture (REDCap) to ensure concealed allocation stratified by site and sex and with variable block sizes. The REDCap randomisation module will generate and conceal the allocation sequence, and research staff enrolling participants will not have access to the sequence prior to assignment. This is an open-label trial in which participants, study staff, investigators and treating clinicians will be aware of treatment assignment after allocation.

### Study interventions

#### Low-dose morphine

Patients will take oral morphine sulphate 5 mg two times per day until as tolerated or until the end of the study (1 year). Two trials suggest that this dose optimally balances efficacy and tolerability.^11 13^ It is also the lowest effective dose to mitigate the risk of dependency and addiction. Participants randomised to morphine will also receive a bowel regimen (polyethylene glycol 3350) to be taken as needed (PRN) for the prevention and management of constipation.

#### Pregabalin

Patients will orally titrate to their maximum tolerated dose up to 150 mg two times per day over 2 weeks using 25 or 50 mg tablets. Similar dosing occurred in a previous trial except their schedule involved quicker up-titration within 6 days.^8^ To mitigate adverse effects due to rapid up-titration, we will allow for a similar maximum tolerated dose, but with up-titration within 2 weeks. Renal-adjusted dosing will follow table 1 based on creatinine clearance.

**Table 1:** Dosing schedule of pregabalin

Phase	Days	CLcr ≥60 mL/min	CLcr 30–60 mL/min	CLcr 15–30 mL/min
Titration	1–7	50 mg AM+50 mg PM	50 mg AM+50 mg PM	25 mg AM+25 mg PM
	8–14	100 mg AM+100 mg PM	100 mg AM+100 mg PM	50 mg AM+50 mg PM
Maintenance	15–21	150 mg AM+150 mg PM	150 mg AM+150 mg PM	75 mg AM+75 mg PM

CLcr, creatinine clearance estimated by the Cockcroft-Gault equation.

Patients will have a telephone visit at 2 weeks with their local study physician to determine their maintenance dose until the end of the study (1 year). If the patient requires stopping of pregabalin for any reason or they reach the end of the study, they should taper their dose over 2 weeks.

#### Cough control therapy

Patients assigned to CCT will attend five 45–60 min virtual sessions delivered by a trained physiotherapist or speech-language pathologist. The structure, based on prior literature^8 9 14^ and our group’s work,^17 18^ includes education, cough suppression strategies, breathing/laryngeal exercises and goal setting (table 2).

**Table 2:** Summary of cough control therapy

Session	Week	Content
1	1	This session will focus on general education about chronic cough (ie, definitions, anatomy and mechanisms), introduction to cough suppression and goal setting. Participants will complete a baseline self-efficacy assessment using a combined measure consisting of the New General Self-Efficacy Scale^34^ and a brief, cough-specific self-efficacy scale developed for this study.^35^ Additionally, an entry interview will explore participants’ lived experiences with chronic cough and their expectations of the programme.
2	2	This session will focus on educating participants about cough triggers, behavioural sensitisation mechanisms and the benefits of non-pharmacological strategies. This session will also include the training and practice of 1–2 cough suppression techniques, with reinforcement of goal-oriented behaviour.
3	3	This session will cover the role of hydration, laryngeal hygiene and breathing exercises to control chronic cough.
4	4	This session will serve as a consolidation session, reviewing all aspects of cough control therapy and exploring the sustainability of the cough control strategies after the intervention.
5	6	This session will evaluate participants’ implementation of cough control strategies in daily life and their progress toward previously established goals. A final self-efficacy reassessment will be conducted, along with an exit interview to qualitatively explore participants’ experiences, barriers to maintenance and perceived impact of the therapy. Interview data will be analysed following a pre-specified protocol using reflexive thematic analysis.^36^

Participants will complete a weekly CCT diary asking them to reflect on the past 7 days and report: (a) how often they used cough control strategies (eg, urge suppression, breathing techniques); (b) in what contexts they applied these strategies (eg, at work, in public, while talking); (c) what barriers or facilitators influenced their ability to use the techniques and (d) the perceived effectiveness of the strategies scored on a 0–10 scale.

We will assess treatment fidelity and therapist adherence using a modified version of the Intervention Fidelity Monitoring Checklist for Trials in Chronic Cough.^22 23^ We will further evaluate participant engagement session-by-session through structured self-report checklists that assess perceived usefulness, understanding and confidence in applying each session’s content.^23^

### Concomitant therapy

Sites will follow the 2020 European Respiratory Society guidelines for the treatment of chronic cough.^6^ During the 6-week randomised phase, use of morphine, pregabalin and non-pharmacological cough interventions outside of the allocated treatment will generally not be permitted to avoid confounding. During this phase, patients will also not be allowed to co-enrol in other clinical trials involving investigational medicinal products for the treatment of RCC/UCC.

Patients who do not respond to their allocated treatment (morphine or pregabalin) will not be permitted to switch to the other neuromodulator or alternative antitussives (eg, gabapentin, amitriptyline, codeine, dextromethorphan) during the 6-week randomisation period. This is to preserve the integrity of treatment allocation and minimise treatment contamination.

Once patients complete the 6-week randomised phase and enter the observational extension, patients can take any concomitant treatments, including switching neuromodulators, engaging in adjunctive therapies or enrolling in other clinical trials of RCC/UCC. We will closely monitor and document therapies taken during the observational extension for up to 1 year.

### Study treatment adherence

We will assess adherence to morphine and pregabalin by self-report and tablet count. The physiotherapist and speech-language pathologist will evaluate adherence to CCT via the number of attended sessions and through weekly adherence diaries.^24^ For participants receiving the PRN bowel regimen, we will record the number of doses taken since the previous visit.

### Study outcomes

#### Primary effectiveness outcome (randomised phase)

For the comparison between morphine versus pregabalin, and CCT versus no CCT, we will evaluate:

1. The change from baseline in 24-hour cough frequency (coughs/hour) measured by the VitaloJAK ambulatory cough monitor at 6 weeks.

#### Secondary effectiveness outcomes (randomised phase)

For the comparison between morphine versus pregabalin, and CCT versus no CCT, we will evaluate:

1. The change from baseline in awake cough frequency (coughs/hour) measured by the VitaloJAK ambulatory cough monitor at 6 weeks.
2. The change from baseline in sleep cough frequency (coughs/hour) measured by the VitaloJAK ambulatory cough monitor at 6 weeks.
3. The change from baseline in cough bouts (defined by inter-cough intervals) measured by the VitaloJAK ambulatory cough monitor at 6 weeks.
4. The change from baseline in cough severity on the McMaster Cough Severity Questionnaire (MCSQ) at 6 weeks.
5. The change from baseline in cough severity on the 100-mm VAS at 6 weeks.
6. The change from baseline in cough-specific quality of life on the LCQ at 6 weeks.
7. The proportion of patients achieving a ≥30% reduction in 24-hour cough frequency at 6 weeks.
8. The proportion of patients achieving a ≥30 mm reduction on the 100-mm CS-VAS at 6 weeks.
9. The proportion of patients achieving a ≥1.3-point improvement on the LCQ at 6 weeks.

#### Secondary effectiveness outcomes (observational extension phase)

1. The change from week 6 (end of randomised phase) in cough severity on the MCSQ at 6 and 12 months.
2. The change from week 6 (end of randomised phase) in cough severity on the 100-mm VAS at 6 and 12 months.
3. The change from week 6 (end of randomised phase) in cough-specific quality of life on the LCQ at 6 and 12 months.

#### Safety outcomes

1. The proportion of patients with any adverse event (AE) for each intervention at 6 weeks, 6 months and 12 months.
2. The proportion of patients with treatment-related AEs for each intervention at 6 weeks, 6 months and 12 months.
3. The proportion of patients with serious adverse events (SAEs) for each intervention at 6 weeks, 6 months and 12 months.
4. The proportion of patients with AEs leading to treatment discontinuation for each intervention at 6 weeks, 6 months and 12 months.

### Visit schedule

Trained research staff will perform all study visits. The study has in-person and telephone visits, with electronic administration of all patient-reported outcome measures on REDCap in English and French. Online supplemental file 4 provides a summary of the visits.

### Outcome measures

#### 24-hour cough monitoring with VitaloJAK

An ambulatory cough monitor (VitaloJAK, Vitalograph, UK) will quantify the number of coughs over a 24-hour period. The VitaloJAK cough monitor consists of an air microphone on the lapel, a chest sensor on the sternum and a battery-operated device around the waist.

The monitor captures audio at 8 kHz and 16-bit waveform format. A study coordinator will transfer recordings to a password-protected and encrypted server, where validated, custom-written software will remove silence and background noises. Outputs include 24-hour, awake and sleep cough frequency expressed as coughs/hour. Cough bouts, defined as more than one cough occurring with inter-cough intervals of 3–10 s,^25^ will be explored as a secondary endpoint.

### Patient-reported outcome measures

#### McMaster Cough Severity Questionnaire

The MCSQ is an 8-item instrument that assesses patients’ perception of cough severity.^26^ The MCSQ has a 1 week recall period and items with 7-point Likert scales. It has two domains (frequency and intensity), and its domain and total scores range from 0 to 6, with higher scores indicating worse cough severity. The MCSQ will serve as a secondary outcome measure and be assessed for its measurement properties.

#### Cough Severity Visual Analogue Scale

The CS-VAS asks patients to mark their cough severity over the last week on a 100-mm VAS ranging from 0 (no cough) to 100 (worst possible cough).^27^ The CS-VAS will serve as a secondary outcome measure and support construct validation of the MCSQ.

#### Leicester Cough Questionnaire

The LCQ is a valid and reliable 19-item instrument that assesses cough impact on quality of life.^28^ The LCQ has a 2-week recall period and items with 7-point Likert scales. It has three domains (physical, psychological and social), and its domain scores range from 1 to 7, with higher scores indicating better cough quality of life. Its total score ranges from 3 (maximal impairment) to 21 (no impairment). Its minimal important differences are 1.3 (LCQ total), 0.8 (physical domain), 0.9 (psychological domain) and 0.8 (social domain).^29^ The LCQ will serve as a secondary outcome measure and support construct validation of the MCSQ.

#### Patient Global Impression of Change and Patient Global Impression of Severity Instruments

The Patient Global Impression of Change (PGIC) assesses patient-reported change at follow-up visits. We will have separate PGIC instruments for cough severity, cough intensity and cough frequency. The PGIC scores changes using a 7-point scale (ie, cough severity: +3=very much better, +2=much better, +1= a little better, 0=no change, −1=a little worse, −2=much worse, −3=very much worse). The PGIC will support construct validation and estimation of the minimal important difference of the MCSQ.

The Patient Global Impression of Severity (PGIS) assesses the patient’s overall perception of the current severity of their cough at each study visit. Separate PGIS instruments will be administered for cough severity, cough intensity and cough frequency. Responses are rated on a 5-point scale ranging from none, mild, moderate, severe and very severe.

#### EuroQol-5 Dimensions-5 Levels

The EuroQol-5 Dimensions-5 Levels (EQ-5D-5L) represents a generic health-related quality of life measure that assesses how a person’s health impacts their quality of life.^30^ The ‘5-Dimension’ refers to the five key areas of health including mobility, self-care, usual activities, pain/discomfort and anxiety/depression. The ‘5-Level’ indicates that each dimension has five levels of severity, ranging from no problems to extreme problems. The EQ-5D-5L will support a cost-effectiveness analysis of the study interventions.

### Data collection

Source documents and electronic case report forms (eCRFs) in REDCap will record all data collected during study visits.

### Withdrawal of study participant

Participants can withdraw from the study at any given time. If a participant stops study treatment, we will strongly encourage participants to attend follow-up visits for collection of outcome data. They will remain eligible for financial compensation based on their continued participation in follow-up assessments, regardless of treatment discontinuation or adherence. If they do not wish to participate in outcome data collection and wish to withdraw from all parts of the study, their anonymised data recorded up to the point of withdrawal will be included in the study analysis. All patient data will be anonymised.

### Sample size

We will enrol 124 patients and randomise them in a two-stage process. First, a 1:1 randomisation will assign patients to low-dose morphine or pregabalin (62 per group). Within each arm, a second independent 1:1 randomisation will assign patients to CCT or no CCT. This will result in four groups of 31 patients each. We powered the study on the 62-patient comparisons (ie, morphine vs pregabalin; CCT vs no CCT) rather than on individual groups. Because we anticipate a smaller effect size for the morphine versus pregabalin contrast, we conservatively based our sample size on this comparison.

The primary outcome is the change from baseline in natural log-transformed 24-hour cough frequency at 6 weeks. Assuming a 30% relative reduction in 24-hour cough frequency^31^ (corresponding to a mean difference of −0.357 units on the log scale), an SD in log coughs/hour of 0.6,^32^ and a 10% dropout, a total sample size of 124 participants (62 per intervention comparison) will provide 80% power at a two-sided alpha of 0.025. To maintain a family-wise type I error rate of 5% across our two co-primary hypotheses (morphine vs pregabalin and CCT vs no CCT), we applied a Bonferroni correction and set the significance level at α=0.025 for each comparison. We performed these calculations using the pwr.t.test function in the pwr package in R studio V.4.3.1 (Posit Software, PBC; Boston, MA, USA).

While our primary interest lies in estimating main effects, the factorial design also permits exploration of potential interactions between treatments. For the sample size calculation, we assumed no significant interaction—that is, that the effect of one intervention is consistent across levels of the other. Under this assumption, we will estimate the effect of morphine versus pregabalin by averaging across CCT and no CCT arms, and the effect of CCT versus no CCT by averaging across morphine and pregabalin arms.

To ensure sufficient participant enrolment, we will monitor recruitment at each site monthly and enact strategies (eg, additional sites) as needed to achieve the target sample size.

## Statistical analysis

### General considerations

A statistician will conduct all analyses. Descriptive statistics (count and percentage for categorical variables and mean or median with SD or IQR for continuous variables, as appropriate) will describe baseline characteristics of enrolled patients.

### Main efficacy analysis at 6 weeks

The primary and responder analyses of all primary and secondary outcomes will occur in the intention-to-treat population, which includes all participants in the group to which they were randomised. Online supplemental file 5 summarises the statistical analysis plan.

### Primary analysis of the primary outcome at 6 weeks

For the primary outcome at 6 weeks, we will use a linear model to estimate changes from baseline in log-transformed 24-hour coughs per hour. The model will include treatment group, site and baseline log-transformed 24-hour cough frequency as fixed effects. Each primary hypothesis (morphine vs pregabalin; CCT vs no CCT) will be tested at a two-sided alpha of 0.025.

We will handle missing data under a missing-at-random assumption using multiple imputation. If ≥15% of outcome data are missing, we will perform a missing-not-at-random (MNAR) sensitivity analysis using a pattern-mixture model. Missing outcomes will be assumed to differ by a fixed offset (eg, 10%) from observed values, and the impact on treatment effects will be examined. Model assumptions (ie, normality, homoscedasticity) will be checked.

Mean changes from baseline in log-transformed 24-hour coughs per hour will be estimated as marginal means with their 95% CIs, and between-group differences (95% CI) will be calculated. To present geometric means of 24-hour coughs per hour for treatment groups at each time point, we will back transform the log-transformed estimates. The percent difference in the change from baseline and 6 weeks between treatment groups will be calculated as 100*(e^diff^−1), where diff represents the between-group difference on the log scale. P values for treatment effects at 6 weeks will be reported.

### Primary analysis of the secondary outcomes at 6 weeks

For continuous secondary outcomes at 6 weeks, we will use linear models or linear mixed-effects models. All models will include site and treatment group as fixed effects. For the MCSQ, CS-VAS and LCQ measured at three timepoints (baseline, 2 weeks and 6 weeks), we will include participant-level intercept as a random effect, and time and the interaction between treatment and time as fixed effects.

We will estimate marginal means (95% CI) at each timepoint. Between-group differences in mean changes from baseline at 6 weeks (95% CI) will be calculated using post hoc contrasts based on the model. Handling of missing data will follow the same approach as the primary outcome.

For secondary outcomes, p values for the treatment effect at week 6 will be reported at the conventional two-sided 0.05 level without adjustment for multiple comparisons. While the trial is powered for and will focus on inferential testing of the primary outcome of 24-hour cough frequency, we recognise that some secondary outcomes may better reflect patient-important benefits. Accordingly, interpretation of the trial findings will consider the overall pattern and consistency of effects across primary and secondary outcomes at 6 weeks.

### Secondary responder analyses at 6 weeks

We will conduct responder analyses to support clinical interpretation of treatment effects analysed on a continuous scale. Responders will include those who achieve a (1) ≥30% reduction from baseline in 24-hour cough frequency^31^; (2) ≥30 mm reduction from baseline on the CS-VAS^33^ and (3) ≥1.3-point improvement from baseline on the LCQ.^29^ We will analyse each responder outcome separately.

For each responder definition, we will report the proportion of responders by treatment group. Generalised linear models with an identity link will estimate between-group absolute risk differences and corresponding 95% CIs. These models will include treatment group and site as fixed effects. Where models fail to converge, risk differences will be estimated using alternative appropriate methods. Handling of missing data will follow the same approach as for the primary outcome.

### Sensitivity analyses

#### Testing the impact of protocol adherence

Anticipating that patients may discontinue treatment due to AEs, we will assess the robustness of the primary analysis at 6 weeks to deviations from the intervention protocol. This sensitivity analysis will occur with the primary outcome and select secondary outcomes in the per-protocol population.

For the morphine versus pregabalin comparison, the per-protocol population will include patients who received ≥80% of the intended study drug over the 6-week period. For the CCT versus no CCT comparison, the per-protocol population will include patients who (1) completed at least four out of the five weekly adherence diaries and (2) reported using at least one cough control strategy in three of those diary entries.

The same linear model or linear mixed-effects model described in the primary analysis will be applied to the per-protocol population.

#### Testing the impact of including site as a random effect

To assess potential clustering by site, we will repeat analyses for the primary and key secondary outcomes using a model that treats site as a random effect. As site represents a stratification variable in the randomisation, we do not anticipate that site-level variation will meaningfully impact treatment effect estimates. However, this sensitivity analysis will account for any unmeasured heterogeneity across sites that may influence outcomes. We will compare estimates with those from the fixed effect model to assess any meaningful differences.

### Subgroup analysis

#### Testing whether the effect of CCT differs depending on neuromodulator treatment

Although not powered to detect interactions, we will conduct an exploratory subgroup analysis evaluating whether the effect of CCT differs depending on the neuromodulator treatment and vice versa.

Linear models will include fixed effects for site, CCT, neuromodulator treatment, their interaction and baseline log 24-hour cough frequency. Secondary outcomes with repeated measures will use mixed-effects models with participant-level intercepts and interaction terms as appropriate. If we observe evidence of a clinically meaningful interaction, treatment effects will be presented stratified by the interacting factor and conclusions will be interpreted accordingly. Interaction effects will be interpreted cautiously as exploratory.

#### Testing whether baseline self-efficacy impacts the effectiveness of CCT

Among participants receiving CCT, we will evaluate whether treatment effectiveness differs by baseline self-efficacy measured with the New General Self-Efficacy Scale (median split). Linear models for the primary outcome will include fixed effects for site, self-efficacy group (low vs high) and baseline log cough frequency. Secondary outcomes with repeated measures will use mixed-effects models with participant-level intercepts and interaction terms.

Post hoc contrasts will estimate the effect of CCT within each self-efficacy group (low vs high) at 6 weeks. These analyses will be exploratory with p values interpreted cautiously.

#### Testing the effect of CCT on self-efficacy

Among participants receiving CCT, a linear model including fixed effects for site, time and baseline self-efficacy will assess changes on the New General Self-Efficacy Scale and the Cough Self-Efficacy Scale.

### Main analyses for observational extension phase

#### Descriptive analyses at 6 and 12 months

We will report the frequency and proportion of patients who continue on their assigned treatment, switch to an alternative treatment, discontinue all treatment (ie, are on no treatment), enrol in another clinical trial and continue to use techniques learnt during the CCT programme. Among those who switch, we will describe the treatments they switch to (eg, morphine, pregabalin, gabapentin, amitriptyline, codeine). For those who switch treatments, discontinue all treatment or enrol in another clinical trial, we will descriptively summarise the reasons (eg, adverse effects, lack of benefit) by initial randomised group and by treatment exposure at the time of the switch or discontinuation. For CCT participants, reasons for not continuing techniques will be documented.

#### Analysis of patient-reported outcomes at 6 and 12 months

Linear mixed-effects models will estimate changes from week 6 (baseline for the extension phase) to 6 and 12 months for the MCSQ, CS-VAS and LCQ. Models will include time, treatment group (time-varying), site and baseline score as fixed effects and participant-level random intercepts.

Treatment group will be defined as a time-varying categorical fixed effect based on the patient’s self-reported treatment at each follow-up visit. The categories will be: (1) on morphine, (2) on pregabalin, (3) on another antitussive, (4) in a clinical trial or (5) not on any treatment.

The primary analysis will be conducted under a missing-at-random assumption using maximum likelihood estimation. If missing outcome data is ≥15%, an MNAR sensitivity analysis will be performed. Marginal means (95% CI) at each timepoint will be estimated. Between-group differences in mean changes from baseline at 6 and 12 months (95% CI) will be calculated using post hoc contrasts. P values for the treatment effect at months 6 and 12 will be reported at the conventional two-sided 0.05 level without adjustment for multiple comparisons. These p values will be interpreted cautiously given the observational and exploratory nature of these analyses.

### Analysis of safety outcomes

We will report all safety outcomes descriptively according to the as-treated population. The as-treated population will include analysing those based on the treatment received, regardless of their initial randomisation. During the randomised phase, AE categories—including participants with any AE, treatment-related AEs, SAEs and discontinuation due to AEs—will be reported descriptively at 6 weeks. AEs occurring in ≥10% of participants in a single group at 6 weeks will be highlighted. In addition, we will specifically monitor and report the incidence of the most common side effects of each study drug. For morphine, this includes constipation and drowsiness/sedation. For pregabalin, this includes dizziness, somnolence and peripheral oedema. Safety outcomes during the observational extension will be similarly reported based on participants’ treatment exposure at 6 and 12 months.

## Study monitoring

### Trial management

The Respiratory Research Lab at McMaster University will act as Study Sponsor, Main Coordinating and Methods Centre, overseeing contracts, ethics submissions, site initiation, training, data quality and finances. It will prepare study materials (standardized operating protocols, manuals, eCRFs), be the point of contact for queries and communicate protocol amendments. The sponsor will oversee study analysis, interpretation, reporting and publication decisions.

### Data management

To protect participant confidentiality, all study data will be anonymised using unique identifiers. Secure files will store personal identifiers separately. Delegated local staff will enter data into REDCap, a secure, web-based, password-protected, encrypted system with role-based access. Validation rules, consistent coding, audits, query resolution and backups will ensure data quality.

### Steering committee and data monitoring committee

The FORTITUDE Trial Steering Committee, comprised of the Principal Investigator, site leads and Coordinating Centre research coordinator, will advise on protocol revisions and provide clinical guidance.

A formal Data Monitoring Committee (DMC) will provide independent oversight of participant safety and trial conduct in accordance with a pre-specified DMC charter. The DMC will consist of one independent statistician and two independent physicians with relevant clinical and trial experience. Given the established safety profiles of the study interventions (morphine, pregabalin and CCT) and the absence of planned interim efficacy analyses, the primary role of the DMC will be to review safety data, protocol adherence and overall trial conduct.

The DMC will review accumulating safety data at regular intervals and may recommend protocol modifications or additional safeguards if safety concerns arise. Monitoring frequency will depend on recruitment rates, unresolved issues and findings from prior reviews, and is expected to occur once or two times annually. The Sponsor and Steering Committee will remain responsible for day-to-day trial oversight and implementation of any DMC recommendations.

### AEs and SAEs

AEs include any adverse health changes during the study and will require documentation in source documents/eCRFs. Local investigators will assess severity, causality and expectedness, following centralised guidance. SAEs include death, life-threatening events, hospitalisation/prolongation, significant disability, congenital anomalies or other medically significant events. SAEs will be recorded in patient hospital notes and source documents/eCRFs and reported to the Sponsor and local research ethics board.

## Ethics and dissemination

Health Canada’s Pharmaceutical Drugs Directorate and the Hamilton Integrated Research Ethics Board (Project ID 18940) have provided study approval. We have pending ethics approvals from the research ethics boards of other participating sites (online supplemental file 1).

Protocol amendments will be documented with rationale and communicated to the sponsor, investigators and sites. If participation or rights change, participants will re-consent.

We will disseminate study findings at international conferences (American Thoracic Society, European Respiratory Society) and through open-access manuscripts in peer-reviewed journals regardless of results. Authorship will follow ICMJE guidelines, acknowledging all sites and personnel. Funders will not influence data handling, analysis or reporting. Reports will adhere to the 2025 CONSORT^19^ and the Factorial Randomised Trials Extension guidelines.^20^

### Data sharing and data access

The Principal Investigator and Steering Committee will review requests for data sharing on an individual basis. After the main publication of the FORTITUDE trial, there may be opportunities to conduct additional analyses on the data collected. Formal requests for additional analyses with a submitted rationale and analysis plan can be made to the Principal Investigator and will be reviewed with the Steering Committee.

### Ancillary and post-trial care

We do not plan specific provisions for ancillary or post-trial care, as all studied interventions are routinely available in clinical practice.

## Discussion

Despite guidelines recommending neuromodulators for the treatment of RCC/UCC and their frequent prescription among clinicians, there remains no head-to-head evidence on the comparative benefits and potential long-term harms of these treatments. FORTITUDE will provide important evidence to guide clinicians on which neuromodulator to prescribe for their patients.

FORTITUDE will also represent the largest trial to evaluate the effectiveness of a virtual CCT programme. Data from utility scores, diaries, attendance, checklists and interviews capturing intervention intensity and patient experience will support cost-effectiveness and acceptability analyses.

A 2×2 factorial design allows simultaneous evaluation of two interventions. Since our objective is to compare low-dose morphine versus pregabalin and virtual CCT versus no CCT, this design provides efficient estimation of main effects with fewer participants than two separate trials. In addition, the trial will study these interventions in combination. While one trial has evaluated pregabalin in combination with speech therapy,^8^ no trials have evaluated non-pharmacological interventions with low-dose morphine.

Although we propose a 2×2 factorial trial with combined interventions, our primary analysis assumes no interaction between neuromodulators and CCT. For separate estimation of main effects, we will assume no interaction between neuromodulators and CCT. As neuromodulators and CCT act through distinct mechanisms—neurobiological versus behavioural modulation of the cough reflex—we believe this assumption may be biologically plausible. The main analysis will independently estimate the effects of neuromodulator treatment (morphine vs pregabalin) and CCT (virtual CCT vs no CCT), with the trial powered accordingly. While we considered a 3×2 factorial design including a placebo group, we opted against this design to keep the study pragmatic and to not withhold active treatment. The primary objective of this trial is to assess the comparative effectiveness of commonly used treatment strategies rather than to estimate efficacy relative to placebo. Because participants will be randomised, any placebo effects are expected to be distributed equally across the active treatment arms and therefore should not bias comparisons between interventions.

Acknowledging that potential synergistic or antagonistic interactions between these treatments may exist, we will perform exploratory subgroup analyses to assess whether the effect of one treatment differs depending on the other. Our study will have limited power, however, to detect statistically significant interactions, and this represents a study limitation. All exploratory analyses investigating interactions will be interpreted with caution and considered hypothesis-generating. Finally, the observational extension phase may introduce confounding and selection bias, limiting causal inferences for long-term outcomes.

## Supplementary material

10.1136/bmjopen-2026-126545 online supplemental file 1

10.1136/bmjopen-2026-126545 online supplemental file 2

10.1136/bmjopen-2026-126545 online supplemental file 3

10.1136/bmjopen-2026-126545 online supplemental file 4

10.1136/bmjopen-2026-126545 online supplemental file 5
