# Supplementary material for: Multicentre, 2×2 factorial, randomised, open-label trial of neuromodulators and cough control therapy for refractory or unexplained chronic cough: the FORTITUDE trial protocol
Source: BMJ Open. 2026 Sep 18;16(9):e126545. doi: 10.1136/bmjopen-2026-126545 (PMC13599969; doi:10.1136/bmjopen-2026-126545)
Supplement: Supplementary file 1 [file bmjopen-16-9-s003.pdf]

PARTICIPANT INFORMATION SHEET/CONSENT FORM

Title of Study: A 2x2 factorial, randomized, open-label trial to evaluate neuromodulators and cough control therapy in patients with refractory or unexplained chronic cough: the FORTITUDE trial

Locally Responsible Investigator and Principal Investigator

1. Dr. Imran Satia, MD PhD, Department of Medicine, Division of Respirology, McMaster University

Local Co-Investigators

2. Dr. Paul O'Byrne, MD, Department of Medicine, Division of Respirology
3. Dr. Wafa Hassan, MD, Department of Medicine, Division of Respirology
4. Dr. Lehana Thabane, PhD, Department of Health Research Methods, Evidence, and Impact
5. Dr. Gordon Guyatt, MD MSc, Department of Health Research Methods, Evidence, and Impact
6. Elena Kum, BMSc, Department of Health Research Methods, Evidence, and Impact

Sponsor: McMaster University, 1280 Main St. West, Hamilton, Ontario, L8S 4K1

Funding Source: Physician Services Incorporated, Canadian Institutes of Health Research

You are being invited to participate in a research study about assessing the effectiveness of treatments for refractory or unexplained chronic cough. Your involvement in this research is strictly voluntary and you will not experience any negative consequences should you wish not to participate.

In order to decide whether or not you want to be a part of this research study, you should understand what is involved and the potential risks and benefits. This form gives detailed information about the research study, which will be emailed to you prior to your first visit. After reading this information sheet, you will be given the opportunity to ask questions about the study. Once you understand the study, you will be asked to sign this form if you wish to participate. Please take your time to make your decision. Feel free to discuss it with your friends and family, or your family physician.

WHY IS THIS RESEARCH BEING DONE?

Refractory or unexplained chronic cough (RCC/UCC) is a common and troublesome condition with no licensed treatments. Two medications commonly prescribed for this condition include low-dose morphine and pregabalin. Both these treatments have shown promise in reducing cough in patients with RCC/UCC. However, it is unclear which of the two works better.

Patients may also receive physiotherapy and speech-language therapy for the treatment of RCC/UCC. However, only a few small studies have looked at its effectiveness.

We are doing the FORTITUDE trial to answer two questions:

1. Which medication—low-dose morphine or pregabalin—is more effective for the treatment of RCC/UCC?
2. How effective is a virtual non-pharmacologic treatment, called cough control therapy (CCT), for the management of RCC/UCC?

Data from our trial will help clinicians and patients choose the most effective and safe treatment option.

WHAT IS THE PURPOSE OF THIS STUDY?

The purpose of the FORTITUDE trial is to evaluate:

1. The effectiveness and tolerability of two commonly prescribed drugs (morphine and pregabalin) for the treatment of RCC/UCC.
2. The effectiveness of a virtual non-pharmacologic intervention (cough control therapy) for the treatment of RCC/UCC.
3. The long-term outcomes of these interventions for patients with RCC/UCC.

FORTITUDE is a randomized trial. That means you have a 25% chance of being assigned to (1) morphine alone (up to 5 mg twice daily), (2) pregabalin alone (up to 150 mg twice daily), (3) morphine (up to 5 mg twice daily) plus virtual cough control therapy, and (4) pregabalin (up to 150 mg twice daily) plus virtual cough control therapy. The study is open label so you and everyone on the study team will know what treatment you have been assigned to.

The results of this clinical trial will inform clinicians on which drug works better in these patients; the effectiveness of a virtual intervention that combines education, motivational interviewing, physiotherapy, and speech-language therapy; and how well they work alone and in combination with each other.

WHAT WILL MY RESPONSIBILITIES BE IF I TAKE PART IN THE STUDY?

The study involves 5 visits over a period of 1 year. Not all study visits will require you to be in person. Most of the study visits will occur over the telephone.

The following is a summary of the procedures involved at each visit:

<u>Visit 0/1 (In Person): Study screening, informed consent, and baseline assessments (1 hour)</u>

- We will review this information letter and consent form, answer any questions that you may have and, if you agree to participate, have you provide informed consent.
- A study physician will review past investigations performed for your cough, take a medical

history, and perform a physical exam to confirm your study eligibility.

- For females of child-bearing potential, you will be asked to perform a urine pregnancy test.
- Some of the blood tests used to check your safety while on study medication may already be ordered as part of your usual medical care. With your permission, we will use those existing results for the study if they are recent and complete. If the treating physician or study team determines a required test is missing, too old, or not suitable for study purposes, an additional blood test will be performed.
- We will ask questions about your cough (i.e., duration, type [dry/productive]).
- We will get you to complete questionnaires related to your cough and quality of life. This should take 10 minutes.
- You will then have a cough monitor fitted on you for the next 24 hours. This is to measure the number of times you cough. You will return this cough monitor by mail with pre-paid postage stamps provided by our team, or in person if you return to the research facility the next day (see *).
- If possible on the same day, you will be randomly assigned to one of four treatment arms: (1) low-dose morphine only, (2) pregabalin only, (3) low-dose morphine plus virtual cough control therapy, or (4) pregabalin plus virtual cough control therapy.
    - Based on your randomized treatment, the study physician will write you a drug prescription to fill out at the pharmacy.
    - If you are assigned to low-dose morphine sulphate therapy, you will receive up to 5 mg given twice daily orally. A study physician will determine your maximum effective and tolerable dose. You will be asked to take this medication for up to 1 year depending on its effectiveness and tolerability.
    - If you are assigned to pregabalin, you will receive up to 150 mg given twice daily orally. A study physician will slowly titrate you up to your maximum effective and tolerable dose. You will be asked to take this medication for up to 1 year depending on its effectiveness and tolerability. If you stop this medication, a study physician will provide instructions on how to wean off the medication gradually.
    - If you are randomized to receive cough control therapy, we will refer you to our physiotherapy and speech-language pathology team who will arrange your sessions. The virtual cough control therapy will consist of five 45- to 60-minute sessions conducted over Zoom over a 6-week period. Sessions 1-4 will occur weekly. Session 5 will occur 2 weeks after Session 4. As part of the cough control therapy, you will be asked to fill out a weekly diary on whether you are using the cough suppression techniques. The cough control therapy sessions will be recorded over Zoom.

*If we need more time to screen you for eligibility and cannot randomize you on the same day, we may ask that you come back to the research facility the next day for randomization. If this is done, we will have you return the 24-hour cough monitor in person.

<u>Visit 2 (Telephone): Assessment of treatment (30 minutes)</u>

- A research coordinator will call you over the telephone two weeks after randomization.
- They will ask you whether you are taking your assigned treatment, how you are doing with your treatment dose, if you have had any adverse effects, and if you have had changes to other medications you are taking.
- They will also guide you to complete questionnaires about your cough on a web application called REDCap. You will require a computer, tablet, or smartphone for this. This should take 10 minutes.

<u>Visit 3 (In Person): Assessment of treatment (1 hour)</u>

- You will come back to our research facility in person six weeks after randomization.
- A research coordinator will ask you whether you are taking your assigned treatment, if you have had any adverse effects, and if you have had changes to other medications you are taking.
- They will fit a cough monitor on you that you will wear over the next 24 hours. This cough monitor will be returned by mail with pre-paid postage stamps provided by our team.
- You will also complete questionnaires about your cough and quality of life. This should take 10 minutes.
- This visit is the end of the randomized phase of the study. You will then enrol into an observational extension phase. From this point forward, you will be allowed to either remain on your randomized treatment, switch to another treatment, take no treatment, or enter another cough clinical trial. This decision will be made by you and the study physician.

<u>Visit 4 (Telephone): Assessment of treatment (30 minutes)</u>
- A research coordinator will call you over the telephone 6 months after randomization.
- They will ask you what treatment you are taking for your cough, if you have had any adverse effects, and if you have had changes to other medications you are taking.
- If you receive cough control therapy, we will ask whether you are still using the cough suppression techniques.
- We will also guide you to complete questionnaires about your cough on a web application called REDCap. You will require a computer, tablet, or smartphone for this. This should take 10 minutes.

<u>Visit 5 (Telephone): Assessment of treatment (30 minutes)</u>
- A research coordinator will call you over the telephone 12 months after randomization.
- They will ask you what treatment you are taking for your cough, if you have had any adverse effects, and if you have had changes to other medications you are taking.
- If you receive cough control therapy, we will ask whether you are still using the cough suppression techniques.
- We will also guide you to complete questionnaires about your cough on a web application called REDCap. You will require a computer, tablet, or smartphone for this. This should take 10 minutes.

Table 1: Summary of study visits and procedures

**Phase**	**Screening**		**Randomized**		**Observational extension**	
**Visit**	**0**	**1***	**2**	**3**	**4**	**5**
**Week**	0	0	2	6	24	52
**Day**	-1	0	14	42	182	365
**Visit Window (days)**	±5	±5	±5	±5	±5	±5
**In-Person/Telephone**	In-Person	In-Person	Telephone	In-Person	Telephone	Telephone
Informed consent	X					
Eligibility criteria	X					
Physical examination	X					
Medical history	X					
Cough history	X					
Blood work***	X					
Urine pregnancy test§§	X					
Randomization	X	X§				
Initiate treatment	X	X§				
Neuromodulator therapy**	--					
Virtual cough control therapy	--					
Adverse events			X	X	X	X

Concomitant medications	X		X	X	X	X
Treatment discontinuation			X	X	X	X
Treatment adherence			X	X	X	X
Vital signs	X			X		
24-hour cough monitoring (VitaloJAK)	X[¶¶]			X[‡‡]		
McMaster Cough Severity Questionnaire (MCSQ)	X		X	X	X	X
Cough Severity Visual Analogue Scale (VAS)	X		X	X	X	X
Leicester Cough Questionnaire (LCQ)	X		X	X	X	X
Patient Global Impression of Change (PGIC)—Cough Severity Instrument				X		
Patient Global Impression of Change (PGIC)—Cough Frequency Instrument				X		
Patient Global Impression of Change (PGIC)—Cough Intensity Instrument				X		
EuroQol-5 Dimensions-5 Levels (EQ-5D-5L)	X			X	X	X

* Visit 1 only done if eligibility assessment, informed consent, randomization, and baseline assessments cannot occur on the same day as Visit 0.

§ Only done at Visit 1 if cannot be done at Visit 0.

** Continue for as long as tolerated or until the end of study (Visit 5).

¶¶ If Visit 0 and 1 are combined, patients will receive instructions to mail back the VitaloJAK cough monitor to their local study site. If Visit 0 and 1 are separate, patients will have the VitaloJAK cough monitor fitted on them at Visit 0 and returned by the patient to their local study site at Visit 1.

‡‡ Patients will receive instructions to mail back the VitaloJAK cough monitor to their local study site.

§§ Only for females of child-bearing potential.

*** Only if required.

Females of child-bearing potential must agree to use a medically approved method of birth control for the duration of the study. All hormonal birth control must be in use for a minimum of three months. Acceptable methods of birth control include: Hormonal contraceptives including oral contraceptives, hormone birth control patch, vaginal contraceptive ring, injectable contraceptives, or hormone implant, double-barrier method, intrauterine devices, non-heterosexual lifestyle or agrees to use contraception if planning on changing to heterosexual partner(s), vasectomy of partner at least 6 months prior to screening. If participants become pregnant, they must immediately inform the study investigator.

POSSIBLE RISKS AND DISCOMFORTS

All medications in this study are currently used in routine clinical practice. However, there are known side effects associated with the treatments we will prescribe in this study.

Morphine sulfate (5 mg twice daily)

Morphine is an opioid medication that has been used safely in clinical care for many years. In this study, it is given at a very low dose (5 mg twice daily). Most people tolerate this dose well, and we will monitor you closely throughout the study.

Common side effects (1 in 10 chance experiences)

These are usually mild, improve over time, and are manageable with simple strategies:

- Constipation
- Nausea or an upset stomach
- Feeling sleepy or tired
- Mild dizziness

We will provide guidance on preventing constipation and managing any stomach upset.

Less common side effects (1 in 100 chance of experiencing)

These are unlikely at this dose, but we still want you to be aware:

- Itching
- Dry mouth
- Headache
- Sweating
- Low blood pressure, which may cause lightheadedness when standing
- Slowed breathing (rare at this dose, especially when taken alone)

Please contact the study team if you feel unusually drowsy, short of breath, or unwell in any way.

Important precautions

- Safe storage and use: This medication is for you only. Do not share it with anyone else, as it could cause serious harm or even death in others. Keep the medication stored securely (for example, in a locked drawer or cabinet) to prevent theft or accidental use by others. Take the tablets exactly as given. Do not crush or chew them.
- Misuse and dependence: All opioids have the potential for misuse or abuse, which can lead to overdose. This risk is very low in the study because of the low dose and close monitoring. With repeated use, the body can develop tolerance (the medication feels weaker over time) and physical dependence (the body adapts to the medication). This is not the same as addiction. If the medication needs to be stopped, the study team will provide guidance on safe discontinuation, including information about possible withdrawal symptoms. A gradual dose reduction (taper) may be used if appropriate, and participants will be advised to contact the study team if withdrawal symptoms or other concerns occur.
- Medication interactions: Morphine can interact with other medicines that cause sedation (such as benzodiazepines or certain sleep aids). The study doctor will review your medications to ensure safety.
- Alcohol: Avoid drinking alcohol while taking morphine, as the combination can increase drowsiness and slow your breathing.
- Driving and activities: Until you know how the medication affects you, avoid driving, operating machinery, or doing activities that require full alertness.
- Seizures: Morphine can lower the seizure threshold. If you have a seizure disorder, please tell the study doctor as this medication may not be appropriate for you.
- Rare skin reaction: A very rare but serious skin reaction called Acute Generalized Exanthematous Pustulosis (AGEP) has been reported with morphine. It usually appears within the first 10 days of starting the medication. Seek immediate medical care if you develop a sudden rash with fever, redness, or many small bumps/pustules.
- Stopping the medication: Even at low doses, stopping suddenly can cause temporary withdrawal symptoms (such as irritability or flu-like feelings). We will guide you if the medication needs to be stopped.
- Breathing conditions: If you have lung disease or sleep apnea, the study physician will take extra care in evaluating your safety.

- Pregnancy: Morphine is not recommended in pregnancy or breastfeeding. You should not become pregnant while taking the study drug. Please inform the study team if you become pregnant or plan to become pregnant.

Pregabalin (up to 150 mg twice daily)

Pregabalin is a medication that is commonly used in routine clinical care for nerve-related symptoms. Most people tolerate it well, especially at the dose used in this study. We will monitor you closely while you are taking it.

Common side effects

These are usually mild and tend to improve as your body gets used to the medication:

- Dizziness
- Feeling tired or drowsy
- Feeling unsteady on your feet

If these occur, they are generally manageable, and the study team can help you with strategies to reduce them.

Less common side effects

These are unlikely, but we still want you to be aware of them:

- Blurred or changed vision
- Swelling of the face, lips, or throat (angioedema)
- Allergic reactions such as rash, hives, or trouble breathing
- Weight gain
- Muscle aches or weakness
- Mood changes
- New or worsening thoughts of self-harm

If you notice any mood changes or distressing thoughts, please contact the study team right away.

Important precautions

- Heart conditions: If you have a heart problem, pregabalin can occasionally cause swelling. The study doctor will review your medical history to ensure it is safe for you.
- Stopping the medication: Pregabalin should not be stopped suddenly. Doing so may cause temporary symptoms like trouble sleeping, nausea, or anxiety. We will give you clear instructions on how to take it safely.
- Medication interactions: Pregabalin can interact with some medicines (such as opioids or benzodiazepines). The study doctor will review all of your medications before you start.
- Alcohol: Because pregabalin can increase drowsiness, you will be asked to avoid alcohol while on the medication.
- Diabetes: Very rarely, pregabalin may affect skin integrity in people with diabetes. Please let us know if you notice any new skin changes.
- Pregnancy: Pregnant or breastfeeding individuals cannot take part in this study. If you become pregnant or plan to become pregnant, please notify the study team right away.
- Animal studies in male reproduction: Pregabalin has demonstrated developmental effects in some animal studies; the relevance of these findings to humans is unknown. To be on the safe side, it might be best if you do not get your partner pregnant while taking the study drug.

Activities

Until you know how pregabalin affects you, please avoid driving, operating machinery, or doing activities that require full alertness.

To mitigate the risks associated with medication side effects, we will monitor you closely at each study visit and through interim check-ins if required. If we identify any adverse effects, we will take appropriate steps including adjusting the dose, stopping treatment, and/or prescribing medication to help manage the side effects (i.e., laxatives for constipation). Your study physician will determine whether it is safe for you to begin taking any one of these drugs. Please tell your study physician if your health status or use of other medications changes during the study.

Cough Control Therapy (CCT)
Cough control therapy involves behavioural training. While not expected to pose significant side effects, some participants may find it time-consuming. You will receive support and clear guidance from trained therapists. They will pace sessions based on your availability and comfort level. The virtual delivery of the CCT is intended to minimize travel and time burden.

Cough Frequency Monitoring
You may experience some inconvenience related to study procedures, such as wearing the VitaloJAK ambulatory cough monitor for 24 hours at two time points in the study. The cough monitor is about the size of a cell phone, and will record all sounds that you are making. This is to count how many times you cough while you are wearing it. The monitor will have a sensor that attaches to your chest, a microphone attached to your collar, and a battery pack that is worn at the waist. The study staff will set up the cough monitor and show you how to wear and use it. The study staff will show you how to remove the monitor at home after the 24-hour recording period ends, or you will return to the study centre and the study staff will help you remove it. There is a very small chance of mild skin irritation from the adhesive. Wearing the VitaloJAK cough monitor also comes with some privacy risks (see section below for details related to privacy).

Other risks and discomforts
Blood tests will be done to check your safety while on study medication, there may be minor discomfort associated with taking a blood sample.

Some study visits will occur over the telephone. To maintain confidentiality, there will be no recording of study visits over the telephone at any time.

While we do not anticipate any incidental health findings, you may report new or unexpected symptoms during the study. If this occurs, we will advise you to consult your health care provider and, with your permission, may share concerns with that treating physician.

Finally, as with any research study, there is a small risk of privacy break. The CCT will be delivered virtually and recorded over Zoom, which is an externally hosted cloud-based service. A link to their privacy policy is available here: https://zoom.us/privacy-and-legal. While the Hamilton Integrated Research Ethics Board has approved using the platform to collect data for the study, there is a small risk of privacy breach for data collected on external servers. Likewise, there is a small risk of privacy breach for all other data collected as part of the study. We will protect your confidentiality through secure data storage, restricted access, and de-identification of data where possible.

IS PARTICIPATION IN THE STUDY VOLUNTARY?

Your participation in this study is voluntary. You may decide to leave the study at any time by communicating this to our Study Coordinator, Sue Beaudin (beaudins@mcmaster.ca), or one of our Study Physicians, Dr. Wafa Hassan (hassaw5@mcmaster.ca) or Dr. Imran Satia (satiai@mcmaster.ca).

Any data collected up until the point of study withdrawal will still be used unless requested otherwise. You can request your data to be removed entirely from the study at any given time prior to study-specific data analysis. After the data have been de-identified and aggregated for data analysis, publication, or presentation, withdrawal of individual data will no longer be possible. You may also decline to answer any question(s) you prefer not to answer.

De-identified study data may be stored and used later on to answer future research questions that arise following the data analysis in this study. That data will not be shared with any other researchers or included in research studies unrelated to the current study.

WHAT INFORMATION WILL BE KEPT PRIVATE?

Your data will not be shared with anyone except with your consent or as required by law. All personal information such as your name, address, email address, and phone number will be removed from the data and will be replaced with a number. A list linking the number with your name will be kept in a secure place, separate from your file. The data with identifying information removed will be securely stored electronically in a password-protected database, accessible only to study personnel for the duration of the study.

Cough recordings captured by the VitaloJAK cough monitor will be labeled with minimal identifying information and sent to Vitalograph, a company based in the Republic of Ireland, EU. Vitalograph will perform cough counting using a semi-automated algorithm that removes most non-cough sounds, including most speech and silences, prior to human annotation of the cough events only.

The recordings made by the cough monitor are analyzed by trained personnel (Vitalograph Cough Analysts), who count the number of times you cough while wearing the monitor. There may be instances when the Vitalograph Cough Analysts hears your conversation - this could include personal conversations. If the Vitalograph Cough Analysts hears information considered to be of serious nature, such as a threat to life or serious criminal activity, they will notify the study doctor for appropriate reporting. Depending on the nature of what was heard, the local research team may need to notify authorities including the police. If this happens, it could have legal consequences for you that would be out of the hands of the investigators, McMaster University, Hamilton Health Sciences, St. Joseph's Healthcare Hamilton, University of Calgary, Laval University, University of Sherbrooke, Western University, or the Sponsor of the study.

You are reminded that you may mute the recording device at any time during the 24-hour period. You can also request that the researchers destroy specific recordings within 3 days of the recording being taken. To do this, please contact the study coordinator, Sue Beaudin (beaudins@mcmaster.ca; 905-525-9140 x22796), or a study physician, Dr. Wafa Hassan (hassaw5@mcmaster.ca; 905-525-9140 x22284) or Dr. Imran Satia (satiai@mcmaster.ca; 905-521-2100 x76228).

It is possible that representatives of the Hamilton Integrated Research Ethics Board (HiREB), this institution and affiliated sites may consult your original research data to check that the information collected for the study is correct and follows proper laws and guidelines. However, no records which

identify you by name or initials will be allowed to leave the hospital. By signing this consent form, you authorize such access.

When the results of the study are published, your name will not be used and no information that discloses your identity will be released or published.

CAN PARTICIPATION IN THE STUDY END EARLY?

If you volunteer to be in this study, you may withdraw at any time and this will in no way affect the quality of care you receive at this institution. Should you wish to withdraw from the study, contact one of the Study Coordinators at the email listed below. You also have the option of removing your data from the study at any given time.

HOW MANY PEOPLE WILL BE IN THIS STUDY?

One hundred and twenty-four patients will be recruited for this study.

WHAT ARE THE POSSIBLE BENEFITS FOR ME AND/OR FOR SOCIETY?

While we cannot guarantee that you will benefit from the treatments prescribed in this study, you will benefit from being reviewed regularly by a respirologist. By participating in the study, you will advance our understanding of which treatments prescribed as part of usual care are most effective and safe for patients. An end of study letter with the study results will be communicated to all participants.

WILL I BE PAID TO PARTICIPATE IN THIS STUDY?

If you agree to take part, you will receive a $320 cheque for your participation in the study. The payment breakdown per study visit is as follows: Visit 0/1: $100, Visit 2: $40, Visit 3: $100, Visit 4: $40, Visit 5: $40. If you leave the study part way, you will receive a pro-rated amount. For any in person visits, you will receive a parking voucher to park at the hospital free of charge.

WILL THERE BE ANY COSTS?

As part of the study, you will be asked to fill a prescription for either low-dose morphine or pregabalin depending on your randomization. As these medications are part of standard care, we expect participants to fill these prescriptions using their existing drug coverage.

Participants who do not have drug coverage may need to cover out-of-pocket costs for filling their prescription at the pharmacy (over 1 year ~$100 for morphine sulphate and ~$400 for pregabalin). Aside from this, your participation in this research project will not involve any additional costs to you or your health care insurer.

IF I HAVE ANY QUESTIONS OR PROBLEMS, WHO CAN I CALL?

If you need assistance or further information, please contact:

Dr. Imran Satia, MD PhD	Work: (905) 521-2100 x76228	Email: satiai@mcmaster.ca
Dr. Wafa Hassan, MD	Work: (905) 525-9140 x22284	Email: hassaw5@mcmaster.ca

Study Coordinator: Sue Beaudin Work: (905) 525-9140 x22796 Email: beaudins@mcmaster.ca

PLEASE TAKE A COPY OF THIS INFORMATION SHEET HOME WITH YOU

CONSENT STATEMENT

Participant:

I have read the preceding information thoroughly. I have had an opportunity to ask questions and all of my questions have been answered to my satisfaction. I agree to participate in this study and be contacted for any future related projects to this study. I understand that I will receive a signed copy of this form.

____

Participant Name   Signature   Date

Person obtaining consent (leave blank, for administrative purposes only):

____

Name, Role in Study   Signature   Date

This study has been reviewed by the Hamilton Integrated Research Ethics Board (HIREB). The HIREB is responsible for ensuring that participants are informed of the risks associated with the research, and that participants are free to decide if participation is right for them. If you have any questions about your rights as a research participant, please call the Office of the Chair, Hamilton Integrated Research Ethics Board at (905) 521-2100 x42013.
