# Supplementary material for: Multicentre, 2×2 factorial, randomised, open-label trial of neuromodulators and cough control therapy for refractory or unexplained chronic cough: the FORTITUDE trial protocol
Source: BMJ Open. 2026 Sep 18;16(9):e126545. doi: 10.1136/bmjopen-2026-126545 (PMC13599969; doi:10.1136/bmjopen-2026-126545)
Supplement: Supplementary file 2 [file bmjopen-2026-126545.reviewer_comments.pdf]

PEER REVIEW HISTORY

BMJ Open publishes all reviews undertaken for accepted manuscripts. Reviewers are asked to complete a checklist review form and are provided with free text boxes to elaborate on their assessment. These free text comments are reproduced below.

ARTICLE DETAILS

Title (Provisional)

A multicentre, 2x2 factorial, randomized, open-label trial of neuromodulators and cough control therapy for refractory or unexplained chronic cough in tertiary cough clinics: the FORTITUDE trial protocol

Authors

Kum, Elena; Hassan, Wafa; Beaudin, Sue; Strong, Geoff; Smith, Kim; Oliveira, Ana; Al-Saiedy, Mustafa; Mancopes, Renata; Grave, Ana Sofia; Bernier, Melanie; Bhalla, Anurag; Brister, Danica L; Côté, Andréanne; Couillard, Simon; Field, Stephen K; Johnston, Faye; Newman, Robert; Popovic, Nada; Kaplan, Alan G.; Brooks, Dina; Guyatt, Gordon; Satia, Imran

---

This article was not externally reviewed at BMJ Open. Protocols that have been independently assessed prior to submission to BMJ Open are usually fast-tracked to publication on the grounds that further substantial changes will not be possible. This independent assessment will usually be external, independent review for both a substantial grant award from a non-commercial or government funder and ethics approval.
