# Supplementary material for: Multicentre, 2×2 factorial, randomised, open-label trial of neuromodulators and cough control therapy for refractory or unexplained chronic cough: the FORTITUDE trial protocol
Source: BMJ Open. 2026 Sep 18;16(9):e126545. doi: 10.1136/bmjopen-2026-126545 (PMC13599969; doi:10.1136/bmjopen-2026-126545)
Supplement: Supplementary file 3 [file bmjopen-16-9-s001.docx]

**Trial Structured Summary**

**Primary Registry and Trial Identifying Number**

NCT07288528

**Date of Registration in Primary Registry**

2025-Dec-17

**Secondary Identifying Numbers**

None

**Sources of Monetary or Material Support**

Physician Services Incorporated, Canadian Institutes of Health Research, the Canada Research Chairs Program, and the Fonds de Recherche du Québec – Santé, Association Pulmonaire du Québec

**Primary Sponsor**

Respiratory Research Lab, McMaster University

**Secondary Sponsor(s)**

None

**Contact for Public Queries**

Dr. Imran Satia

Address: 1200 Main St West, Hamilton, ON, Canada L8N 3Z5; Tel: 905-521-2100 x76645; Email: satiai@mcmaster.ca

**Contact for Scientific Queries**

Dr. Imran Satia

Address: 1200 Main St West, Hamilton, ON, Canada L8N 3Z5; Tel: 905-521-2100 x76645; Email: satiai@mcmaster.ca

**Public Title**

Randomized trial to evaluate neuromodulators and cough control therapy in patients with refractory or unexplained chronic cough

**Scientific Title**

A 2x2 factorial, randomized, open-label trial to evaluate neuromodulators and cough control therapy in patients with refractory or unexplained chronic cough: the FORTITUDE trial

**Countries of Recruitment**

Canada

**Health Condition(s) or Problem(s) Studied**

Refractory or unexplained chronic cough

**Interventions**

(1) Morphine sulphate (up to 5 mg twice daily)
(2) Pregabalin (up to 150 mg twice daily)
(3) Morphine sulphate (up to 5 mg twice daily) plus cough control therapy
(4) Pregabalin (up to 150 mg twice daily) plus cough control therapy

**Key Inclusion and Exclusion Criteria**

Eligible patients will include adults (≥18 years old) with either:

(1) Refractory chronic cough (RCC), defined as having one or more identifiable causes of cough (asthma, upper airway disease, and/or GERD), but with previous trials of treatment targeting the underlying condition leading to no cough resolution; OR
(2) Unexplained chronic cough (UCC), defined as having a cough lasting >8 weeks with (i) a normal chest X-ray/CT in the last 5 years and since the onset of chronic cough; (ii) FEV_1_ and FVC ≥80% predicted or >lower limit of normal (LLN); (iii) FEV_1_/FVC ≥0.7 or >LLN; (iv) no evidence of asthma; and (vi) no regular symptoms of nasal/reflux disease.

We will exclude patients meeting any of the following criteria:

(1) Those who, in the opinion of the investigator, have previously tried low-dose morphine, pregabalin, and/or a CCT with full intervention fidelity for the treatment of RCC/UCC;
(2) Are currently taking morphine or pregabalin for the treatment of any indication (i.e., RCC/UCC or chronic pain);*
(3) Are currently taking other centrally-acting medication (i.e., gabapentin, amitriptyline, dextromethorphan) for the treatment of any indication (i.e., RCC/UCC, seizures, or depression);*
(4) Have a history of opioid or substance abuse that, in the opinion of the investigator, may interfere with the conduct of the study;
(5) Are a current or ex-smoker with a >20 pack-year history or abstinence <6 months;
(6) Have cough due to angiotensin-converting enzyme inhibitor use;
(7) Have symptoms of an upper respiratory infection within the last month that have not resolved;
(8) Had an asthma exacerbation within the last month that requires an increase or start of an oral corticosteroid;
(9) Have other primary pulmonary disorders, including pulmonary embolism, pulmonary hypertension, lung cancer, cystic fibrosis, radiologically-proven emphysema, interstitial lung disease, or severe bronchiectasis;
(10) Have language, memory, cognitive, or psychiatric issues that may prevent optimal participation;
(11) Have creatinine clearance <15 mL/min, including individuals with end-stage renal disease who require hemodialysis or peritoneal dialysis;
(12) Have any other condition that, in the opinion of the qualified investigator, may adversely affect the participant’s ability to complete the study or its measures or pose significant risk to the participant;
(13) Have participated in another clinical trial of an investigational medicinal product within 30 days;
(14) Pregnant or breastfeeding; or
(15) Females of child-bearing potential who:
a. Do not agree to use a medically approved method of birth control for the duration of the study. All hormonal birth control must have been in use for a minimum of 3 months.
b. Acceptable methods of birth control include oral contraceptives, hormone birth control patch, vaginal contraceptive ring, injectable contraceptives, or hormone implant, double-barrier method, intrauterine devices, non-heterosexual lifestyle or agrees to use contraception if planning on changing to heterosexual partner(s), vasectomy of partner at least 6 months prior to screening.
c. Females who are not of child-bearing potential will be defined as females who have undergone a sterilization procedure (e.g., hysterectomy, bilateral oophorectomy, bilateral tubal ligation, complete endometrial ablation) or have been post-menopausal for at least 1 year prior to screening.

* Participants taking any of the above medications at baseline will only be eligible if they are willing and medically able to safely discontinue them at least 2 weeks prior to the first study visit. Eligibility will be confirmed by the local study investigator prior to enrolment.

**Study Type**

Type of study: Interventional

Method of allocation: Randomized

Masking: Open-label

Assignment: Factorial

Phase: 4

**Date of First Enrollment**

TBD

**Sample Size**

124

**Recruitment Status**

Pending: participants are not yet being recruited or enrolled at any site

**Primary Outcome(s)**

The change from baseline in 24-hour cough frequency (coughs/hour) measured by the VitaloJAK ambulatory cough monitor at 6 weeks.

**Key Secondary Outcome(s)**

(1) The change from baseline in awake cough frequency (coughs/hour) measured by the VitaloJAK ambulatory cough monitor at 6 weeks.
(2) The change from baseline in sleep cough frequency (coughs/hour) measured by the VitaloJAK ambulatory cough monitor at 6 weeks.
(3) The change from baseline in cough bouts (defined by inter-cough intervals) measured by the VitaloJAK ambulatory cough monitor at 6 weeks.
(4) The change from baseline in cough severity on the McMaster Cough Severity Questionnaire at 6 weeks.
(5) The change from baseline in cough severity on the 100-mm visual analogue scale at 6 weeks.
(6) The change from baseline in cough-specific quality of life on the Leicester Cough Questionnaire at 6 weeks.
(7) Proportion of patients achieving a ≥30% reduction in 24-hour cough frequency at 6 weeks.
(8) Proportion of patients achieving a ≥30-mm reduction on the 100-mm cough severity visual analogue scale at 6 weeks.
(9) Proportion of patients achieving a ≥1.3-point improvement on the Leicester Cough Questionnaire at 6 weeks.
(10) The change from week 6 (end of randomized phase) in cough severity on the McMaster Cough Severity Questionnaire at 6 and 12 months.
(11) The change from week 6 (end of randomized phase) in cough severity on the 100-mm visual analogue scale at 6 and 12 months.
(12) The change from week 6 (end of randomized phase) in cough-specific quality of life on the Leicester Cough Questionnaire at 6 and 12 months.

Safety outcomes

(1) The proportion of patients with any adverse event for each intervention at 6 weeks, 6 months, and 12 months.
(2) The proportion of patients with treatment-related adverse events for each intervention at 6 weeks, 6 months, and 12 months.
(3) The proportion of patients with serious adverse events for each intervention at 6 weeks, 6 months, and 12 months.
(4) The proportion of patients with adverse events leading to discontinuation for each intervention at 6 weeks, 6 months, and 12 months.

**Ethics Review**

Approved by the Hamilton Integrated Research Ethics Board.

Ethical approval is pending from the following research ethics boards:

Comité d'éthique de la recherche du Centre intégré universitaire de santé et de services sociaux de l'Estrie–Centre hospitalier universitaire de Sherbrooke;

Comités d’éthique de la recherche avec des êtres humains de l’Université Laval;

Conjoint Health Research Ethics Board, University of Calgary; and

Office of Human Research Ethics, Western University.
