# Supplementary material for: Multicentre, 2×2 factorial, randomised, open-label trial of neuromodulators and cough control therapy for refractory or unexplained chronic cough: the FORTITUDE trial protocol
Source: BMJ Open. 2026 Sep 18;16(9):e126545. doi: 10.1136/bmjopen-2026-126545 (PMC13599969; doi:10.1136/bmjopen-2026-126545)
Supplement: Supplementary file 5 [file bmjopen-16-9-s004.docx]

**Study visits and procedures**

| **Phase** | **Screening** | | **Randomized phase** | | **Observational extension** | |
| --- | --- | --- | --- | --- | --- | --- |
| **Visit** | **0** | **1*** | **2** | **3** | **4** | **5** |
| **Week** | 0 | 0 | 2 | 6 | 24 | 52 |
| **Day** | -1 | 0 | 14 | 42 | 182 | 365 |
| **Visit Window (days)** | ±5 | ±5 | ±5 | ±5 | ±5 | ±5 |
| **In-Person/Telephone** | In-Person | In-Person | Telephone | In-Person | Telephone | Telephone |
| Informed consent | X |  |  |  |  |  |
| Eligibility criteria | X |  |  |  |  |  |
| Review of chest X-ray/CT from the past 5 years^¶^ | X |  |  |  |  |  |
| Review of spirometry with post-bronchodilator reversibility from the past 6 months | X |  |  |  |  |  |
| Review of methacholine challenge from the past 6 months^‡^ | X |  |  |  |  |  |
| Review of sputum induction with differential cell count from the past 6 months^†^ | X |  |  |  |  |  |
| Review of complete blood count (CBC) from the past 6 months | X |  |  |  |  |  |
| Review of serum creatinine from the past 3 months | X |  |  |  |  |  |
| Physical examination | X |  |  |  |  |  |
| Medical history | X |  |  |  |  |  |
| Cough history | X |  |  |  |  |  |
| Urine pregnancy test or serum beta-hCG^††^ | X |  |  |  |  |  |
| Randomization | X | X^§^ |  |  |  |  |
| Initiate treatment | X | X^§^ |  |  |  |  |
| Neuromodulator therapy** | ------------------------------------------------------------------------------------------------------------------------- | | | | | |
| Virtual cough control therapy | --------------------------------------------------------------------------------- | | | |  | |
| Adverse events | X | X | X | X | X | X |
| Concomitant medications | X |  | X | X | X | X |
| Treatment discontinuation |  |  | X | X | X | X |
| Treatment adherence |  |  | X | X | X | X |
| Vital signs | X |  |  | X |  |  |
| 24-hour cough monitoring (VitaloJAK) | X^¶¶^ |  |  | X^‡‡^ |  |  |
| McMaster Cough Severity Questionnaire (MCSQ) | X |  | X | X | X | X |
| Cough Severity Visual Analogue Scale (CS-VAS) | X |  | X | X | X | X |
| Leicester Cough Questionnaire (LCQ) | X |  | X | X | X | X |
| Patient Global Impression of Change & Severity (PGIC & PGIS)—Cough Severity Instrument |  |  |  | X |  |  |
| Patient Global Impression of Change & Severity (PGIC & PGIS)—Cough Frequency Instrument |  |  |  | X |  |  |
| Patient Global Impression of Change & Severity (PGIC & PGIS)—Cough Intensity Instrument |  |  |  | X |  |  |
| EuroQol-5 Dimensions-5 Levels (EQ-5D-5L) | X |  |  | X | X | X |
| Weekly adherence diary^§§^ | --------------------------------------------------------------------------------- | | | |  |  |
| Therapist treatment fidelity checklist at each session*** | --------------------------------------------------------------------------------- | | | |  |  |
| Participant treatment fidelity checklist at each session^§§^ | --------------------------------------------------------------------------------- | | | |  |  |
| New General Self-Efficacy Scale^§§^ | X |  |  | X |  |  |
| Cough Self-Efficacy Scale^§§^ | X |  |  | X |  |  |
| CCT interviews^§§^ | X |  |  | X |  |  |

* Visit 1 only done if eligibility assessment, informed consent, randomization, and baseline assessments cannot occur on the same day as Visit 0.

^¶^ If not performed within the 5 years before Visit 1 and following the onset of chronic cough, a chest X-ray/CT may be obtained prior to Visit 1. Chest X-ray/CT can be repeated as clinically warranted at the discretion of the investigator.

^‡^ Only if clinically warranted.

^†^ Only if clinically warranted and where available.

^§^ Only done at Visit 1 if cannot be done at Visit 0.

** Continue for as long as tolerated or until the end of study (Visit 5).

^¶¶^ If Visit 0 and 1 are combined, patients will receive instructions to mail back the VitaloJAK cough monitor to their local study site. If Visit 0 and 1 are separate, patients will have the VitaloJAK cough monitor fitted on them at Visit 0 and returned by the patient to their local study site at Visit 1.

^‡‡^ Patients will receive instructions to mail back the VitaloJAK cough monitor to their local study site.

^††^ Only in females of child-bearing potential.

^§§^ Only in individuals receiving virtual cough control therapy.

*** Completed by therapists delivering virtual cough control therapy.
