# Supplementary material for: Multicentre, 2×2 factorial, randomised, open-label trial of neuromodulators and cough control therapy for refractory or unexplained chronic cough: the FORTITUDE trial protocol
Source: BMJ Open. 2026 Sep 18;16(9):e126545. doi: 10.1136/bmjopen-2026-126545 (PMC13599969; doi:10.1136/bmjopen-2026-126545)
Supplement: Supplementary file 6 [file bmjopen-16-9-s005.docx]

**Summary of statistical analysis plan for the 6-week randomized phase**

| **Objective** | **Outcome** | **Research hypothesis** | **Analysis method** |
| --- | --- | --- | --- |
| **Primary analysis of the primary outcome** | | | |
| To determine the effect of morphine versus pregabalin on the outcome | Change in 24-hour cough frequency (log-transformed) at 6 weeks | Morphine will reduce 24-hour cough frequency compared to pregabalin | Linear model including fixed effects for treatment, site, and baseline log 24-hour cough frequency |
| To determine the effect of virtual CCT versus no CCT on the outcome |  | Virtual CCT will reduce 24-hour cough frequency compared to no CCT |  |
| **Primary analysis of the secondary outcomes** | | | |
| To determine the effect of morphine versus pregabalin on the outcome | Change in awake cough frequency (log-transformed) at 6 weeks | Morphine will reduce awake cough frequency compared to pregabalin | Linear model including fixed effects for treatment, site, and baseline log awake cough frequency |
|  | Change in sleep cough frequency (log-transformed) at 6 weeks | Morphine will have no effect on sleep cough frequency compared to pregabalin | Linear model including fixed effects for treatment, site, and baseline log sleep cough frequency |
|  | Change in cough bouts (log-transformed) at 6 weeks | Morphine will reduce cough bouts compared to pregabalin | Linear model including fixed effects for treatment, site, and baseline log cough bout frequency |
|  | Change in the McMaster Cough Severity Questionnaire (MCSQ)—Total Score at 6 weeks | Morphine will improve cough severity on the MCSQ compared to pregabalin | Linear mixed-effects model including fixed effects for site, treatment, time, their interaction, and baseline MCSQ score, with participant-level intercept specified as a random effect |
|  | Change in the 100-mm cough severity visual analogue scale (CS-VAS) at 6 weeks | Morphine will improve cough severity on the CS-VAS compared to pregabalin | Linear mixed-effects model including fixed effects for site, treatment, time, their interaction, and baseline CS-VAS score, with participant-level intercept specified as a random effect |
|  | Change in the Leicester Cough Questionnaire (LCQ)—Total Score at 6 weeks | Morphine will improve cough quality of life on the LCQ compared to pregabalin | Linear mixed-effects model including fixed effects for site, treatment, time, their interaction, and baseline LCQ score, with participant-level intercept specified as a random effect |
| To determine the effect of virtual CCT versus no CCT on the outcome | Change in awake cough frequency (log-transformed) at 6 weeks | CCT will reduce awake cough frequency compared to no CCT | Linear model including fixed effects for treatment, site, and baseline log awake cough frequency |
|  | Change in sleep cough frequency (log-transformed) at 6 weeks | CCT will have no effect on sleep cough frequency compared to no CCT | Linear model including fixed effects for treatment, site, and baseline log sleep cough frequency |
|  | Change in cough bouts (log-transformed) at 6 weeks | CCT will reduce cough bouts compared to no CCT | Linear model including fixed effects for treatment, site, and baseline log cough bout frequency |
|  | Change in the MCSQ—Total Score at 6 weeks | CCT will improve cough severity on the MCSQ compared to no CCT | Linear mixed-effects model including fixed effects for site, treatment, time, their interaction, and baseline MCSQ score, with participant-level intercept specified as a random effect |
|  | Change in the CS-VAS at 6 weeks | CCT will improve cough severity on the CS-VAS compared to no CCT | Linear mixed-effects model including fixed effects for site, treatment, time, their interaction, and baseline CS-VAS score, with participant-level intercept specified as a random effect |
|  | Change in the LCQ—Total Score at 6 weeks | CCT will improve cough quality of life on the LCQ compared to no CCT | Linear mixed-effects model including fixed effects for site, treatment, time, their interaction, and baseline LCQ score, with participant-level intercept specified as a random effect |
| **Responder analyses** | | | |
| To determine the effect of morphine versus pregabalin on the outcome | Risk difference for achieving a ≥30% reduction in 24-hour cough frequency (log-transformed) at 6 weeks | A greater proportion of participants receiving morphine will achieve a response in 24-hour cough frequency compared with those receiving pregabalin | Linear model with identity link including fixed effects for treatment and site |
|  | Risk difference for achieving a ≥30-mm reduction on the CS-VAS at 6 weeks | A greater proportion of participants receiving morphine will achieve a response on the CS-VAS compared with those receiving pregabalin |  |
|  | Risk difference for achieving a ≥1.3-point improvement on the LCQ at 6 weeks | A greater proportion of participants receiving morphine will achieve a response on the LCQ compared with those receiving pregabalin |  |
| To determine the effect of virtual CCT versus no CCT on the outcome | Risk difference for achieving a ≥30% reduction in 24-hour cough frequency (log-transformed) at 6 weeks | A greater proportion of participants receiving CCT will achieve a response in 24-hour cough frequency compared with those receiving no CCT |  |
|  | Risk difference for achieving a ≥30-mm reduction on the CS-VAS at 6 weeks | A greater proportion of participants receiving CCT will achieve a response on the CS-VAS compared with those receiving no CCT |  |
|  | Risk difference for achieving a ≥1.3-point improvement on the LCQ at 6 weeks | A greater proportion of participants receiving CCT will achieve a response on the LCQ compared with those receiving no CCT |  |
| **Sensitivity analyses** | | | |
| To determine the effect of morphine versus pregabalin under optimal protocol adherence (those who received ≥80% of the intended study drug over the 6-week period, regardless of CCT adherence) | Change in 24-hour cough frequency (log-transformed) at 6 weeks | Protocol adherence will change the effect of morphine versus pregabalin on 24-hour cough frequency | Linear model including fixed effects for treatment, site, and baseline log 24-hour cough frequency |
|  | Change in the MCSQ—Total Score at 6 weeks | Protocol adherence will change the effect of morphine versus pregabalin on the MCSQ | Linear mixed-effects model including fixed effects for site, treatment, time, their interaction, and baseline MCSQ score, with participant-level intercept specified as a random effect |
|  | Change in the LCQ—Total Score at 6 weeks | Protocol adherence will change the effect of morphine versus pregabalin on the LCQ | Linear mixed-effects model including fixed effects for site, treatment, time, their interaction, and baseline LCQ score, with participant-level intercept specified as a random effect |
| To determine the effect of virtual CCT vs no CCT under optimal protocol adherence (those who (1) complete at least four out of the five weekly adherence diaries and (2) report using at least one cough control strategy in three of those diary entries) | Change in 24-hour cough frequency (log-transformed) at 6 weeks | Protocol adherence will change the effect of CCT versus no CCT on 24-hour cough frequency | Linear model including fixed effects for treatment, site, and baseline log 24-hour cough frequency |
|  | Change in the MCSQ—Total Score at 6 weeks | Protocol adherence will change the effect of CCT versus no CCT on the MCSQ | Linear mixed-effects model including fixed effects for site, treatment, time, their interaction, and baseline MCSQ score, with participant-level intercept specified as a random effect |
|  | Change in the LCQ—Total Score at 6 weeks | Protocol adherence will change the effect of CCT versus no CCT on the LCQ | Linear mixed-effects model including fixed effects for site, treatment, time, their interaction, and baseline LCQ score, with participant-level intercept specified as a random effect |
| If ≥15% of outcome data is missing, to determine the effect of morphine versus pregabalin under a missing not at random (MNAR) assumption | Change in 24-hour cough frequency (log-transformed) at 6 weeks | Missing data will change the effect of morphine versus pregabalin on 24-hour cough frequency | Pattern-mixture linear model with delta adjustment, including fixed effects for treatment, site, and baseline log 24-hour cough frequency |
|  | Change in the MCSQ—Total Score at 6 weeks | Missing data will change the effect of morphine versus pregabalin on the MCSQ | Pattern-mixture linear mixed-effects model with delta adjustment, including fixed effects for site, treatment, time, their interaction, and baseline MCSQ score, with participant-level intercept specified as a random effect |
|  | Change in the LCQ—Total Score at 6 weeks | Missing data will change the effect of morphine versus pregabalin on the LCQ | Pattern-mixture linear mixed-effects model with delta adjustment, including fixed effects for site, treatment, time, their interaction, and baseline LCQ score, with participant-level intercept specified as a random effect |
| If ≥15% of outcome data is missing, to determine the effect of virtual CCT versus no CCT under a missing not at random (MNAR) assumption | Change in 24-hour cough frequency (log-transformed) at 6 weeks | Missing data will change the effect of CCT versus no CCT on 24-hour cough frequency | Pattern-mixture linear model with delta adjustment, including fixed effects for treatment, site, and baseline log 24-hour cough frequency |
|  | Change in the MCSQ—Total Score at 6 weeks | Missing data will change the effect of CCT versus no CCT on the MCSQ | Pattern-mixture linear mixed-effects model with delta adjustment, including fixed effects for site, treatment, time, their interaction, and baseline MCSQ score, with participant-level intercept specified as a random effect |
|  | Change in the LCQ—Total Score at 6 weeks | Missing data will change the effect of CCT versus no CCT on the LCQ | Pattern-mixture linear mixed-effects model with delta adjustment, including fixed effects for site, treatment, time, their interaction, and baseline LCQ score, with participant-level intercept specified as a random effect |
| To determine whether including site as a random effect impacts the effect of morphine versus pregabalin | Change in 24-hour cough frequency (log-transformed) at 6 weeks | Site as a random effect will not change the effect of morphine versus pregabalin on 24-hour cough frequency | Linear model including fixed effects for treatment and baseline log 24-hour cough frequency, and site as a random effect |
|  | Change in the MCSQ—Total Score at 6 weeks | Site as a random effect will not change the effect of morphine versus pregabalin on the MCSQ | Linear mixed-effects model including fixed effects for treatment, time, their interaction, and baseline MCSQ score, with participant-level intercept and site specified as random effects |
|  | Change in the LCQ—Total Score at 6 weeks | Site as a random effect will not change the effect of morphine versus pregabalin on the LCQ | Linear mixed-effects model including fixed effects for treatment, time, their interaction, and baseline LCQ score, with participant-level intercept and site specified as random effects |
| To determine whether including site as a random effect impacts the effect of virtual CCT versus no CCT | Change in 24-hour cough frequency (log-transformed) at 6 weeks | Site as a random effect will not change the effect of CCT versus no CCT on 24-hour cough frequency | Linear model including fixed effects for treatment and baseline log 24-hour cough frequency, and site as a random effect |
|  | Change in the MCSQ—Total Score at 6 weeks | Site as a random effect will not change the effect of CCT versus no CCT on the MCSQ | Linear mixed-effects model including fixed effects for treatment, time, their interaction, and baseline MCSQ score, with participant-level intercept and site specified as random effects |
|  | Change in the LCQ—Total Score at 6 weeks | Site as a random effect will not change the effect of CCT versus no CCT on the LCQ | Linear mixed-effects model including fixed effects for treatment, time, their interaction, and baseline LCQ score, with participant-level intercept and site specified as random effects |
| **Subgroup analyses** | | | |
| To determine whether the effect of CCT differs depending on neuromodulator treatment (morphine versus pregabalin) | Change in 24-hour cough frequency (log-transformed) at 6 weeks | There may be a synergistic effect between CCT and morphine or CCT and pregabalin in terms of 24-hour cough frequency | Linear model including site, treatment, baseline log 24-hour cough frequency, and a CCT x neuromodulator interaction term; post hoc contrasts to estimate the effect of CCT within each neuromodulator group |
|  | Change in the MCSQ—Total Score at 6 weeks | There may be a synergistic effect between CCT and morphine or CCT and pregabalin in terms of cough severity on the MCSQ | Linear mixed-effects model including time, site, treatment, neuromodulator group, baseline MCSQ score, and all two- and three-way interactions among time, treatment, and neuromodulator, with a participant-level random intercept; post hoc contrasts will estimate the effect of CCT within each neuromodulator group |
|  | Change in the LCQ—Total Score at 6 weeks | There may be a synergistic effect between CCT and morphine or CCT and pregabalin in terms of cough quality of life on the LCQ | Linear mixed-effects model including time, site, treatment, neuromodulator group, baseline LCQ score, and all two- and three-way interactions among time, treatment, and neuromodulator, with a participant-level random intercept; post hoc contrasts will estimate the effect of CCT within each neuromodulator group |
| To determine whether response to CCT differs by baseline self-efficacy measured on the New General Self-Efficacy Scale | Change in 24-hour cough frequency (log-transformed) at 6 weeks | Patients with greater self-efficacy at baseline are more likely to benefit from CCT in terms of 24-hour cough frequency | Linear model including site, baseline log 24-hour cough frequency, and baseline self-efficacy group (low vs high); post hoc contrasts to estimate the effect of CCT within each self-efficacy group |
|  | Change in the MCSQ—Total Score at 6 weeks | Patients with greater self-efficacy at baseline are more likely to benefit from CCT in terms of cough severity on the MCSQ | Linear mixed-effects model including time, site, baseline self-efficacy group (low vs high), baseline MCSQ score, and the interaction between time and self-efficacy group, with a participant-level random intercept; post hoc contrasts will estimate the effect of CCT within each self-efficacy group |
|  | Change in the LCQ—Total Score at 6 weeks | Patients with greater self-efficacy at baseline are more likely to benefit from CCT in terms of cough quality of life on the LCQ | Linear mixed-effects model including time, site, self-efficacy group (low vs high), baseline LCQ score, and the interaction between time and self-efficacy group, with a participant-level random intercept; post hoc contrasts will estimate the effect of CCT within each self-efficacy group |
| To determine the effect of CCT on self-efficacy | Change in the New General Self-Efficacy Scale (NGSES)—Total Scale at 6 weeks | CCT will improve self-efficacy on the NGSES at 6 weeks compared to baseline | Linear model including site, time, and baseline NGSES score |
|  | Change in the Cough Self-Efficacy Scale (CSES) at 6 weeks | CCT will improve self-efficacy on the CSES at 6 weeks compared to baseline | Linear model including site, time, and baseline CSES score |
